# Colemans Dental Surgery and Pathology

**Published:** 1882-12

**Authors:** 


### BIBLIOGRAPHICAL.

---

*Coleman's Dental Surgery and Pathology.* By Alfred Coleman, L. R. C. P. Dental Lecturer St. Bartholomeus Hospital, etc. etc. etc.

Thoroughly revised and adapted to the use of American Students and Practioners. By Thos. Stellwagen M., A., M., D., D. D. S., Professor of Physiology in Philadelphia Dental College; Publishers, Henry C. Leas' Son & Co., Philadelphia, 1882.

The Editor of this work deserves great credit for the labor he has bestowed upon it to adapt it to the American Student of Dentistry. One hundred and twelve additional illustrations add to the value of the work, together with the important additions of text covering about one hundred pages. In a work of such a size, many subjects are necessarily treated in a brief manner, but the descriptions are lucid and of a character which makes it an important addition to the text books of dental science. The chapter on irregularities while not as voluminous as in other works, contains much valuable instruction, and a description of the latest appliances which have been suggested for the correction of deviating teeth. The chapter on methods of crowning teeth is also a valuable one, the recent improvements, being fully described.

The appearance and style in which this work is published is worthy of the reputation of the well known Publishers.

